# The Comparison of Biomechanical Volar and Dorsal Plating in Distal Part Radius Fractures; a Finite Element Analysis Study

**DOI:** 10.30476/BEAT.2020.86681

**Published:** 2021-01

**Authors:** Ali Ghaem-Maghami, Ehsan Fallah, Hamid Namazi, Mohammad-Taghi Karimi, Seyed Iman Hosseini

**Affiliations:** 1 *Department of Orthopedic Surgery, School of Medicine, AJA University of Medical Sciences, Tehran, Iran*; 2 *Bone and Joint Diseases Research Center, Shiraz University of Medical Sciences, Shiraz, Iran*; 3 *Rehabilitation Sciences Research Center, Shiraz University of Medical Sciences, Shiraz Iran*; 4 *Department of Mechanical and Aerospace Engineering, Shiraz University of Technology, Shiraz, Iran*

**Keywords:** Radius Fracture, Volar, Dorsal, Stress, Displacement, Finite Element Analysis

## Abstract

**Objective:**

To compare the stability of the radius stabilized fractured parts by volar and dorsal planting based on modeling approach.

**Methods:**

Ten forearm models were created based on Computed Tomography (CT) Scan images by using of Mimics software. The distal part fracture of radius was induced in the models. The stress were developed and implanted in various parts of the bone and and their displacement were evaluated in volar and dorsal inserted implants.

**Results:**

The results of this study showed that the stress developed in screws, implant and bony parts differed significantly between volar and dorsal plate conditions. The displacement of implant and bony parts in volar plating was more than dorsal plating (*p*=0.05). However, the screws displacement in dorsal plating significantly increased compared to volar plating.

**Conclusion:**

The stress developed in dorsal and volar implants is not too high to fail the structure. However, it seems that the irritation of soft tissue and tendon would be less in volar inserted implant than dorsal implant. It is recommended to use valor plating to be a good approach for stabilizing the distal part fracture of radius.

## Introduction

Distal radial fractures are the most common fractures encountered by an orthopedic surgeon in his or her professional life [1, 2]. These fractures are caused either by high energy traumas or sport injury in younger patients or by low energy traumas in the elderly. Most distal radial fractures have dorsal comminution and are dorsally displaced and angulated [3].

Different treatment methods are introduced in fractures such as close reduction and casting, close reduction and percutaneous pinning, close reduction and external fixation with or without plating, open reduction and plating or a combination of mentioned treatments above. The treatment choice picked up is depending on comminution, fracture type, bone density, accessible equipment and surgeon experience [4]. 

There is no definite evidence to prove anyone to be more effective than others although different treatment methods exist. Any other skeletal fracture has not such treatment controversy [5]. The purpose of treatment should be anatomic reduction, stable fixation and early rehabilitation and mobilization [6]. Recently, American Orthopedic Association determined radial shortening more than 3 millimeters, dorsal tilt more than 10 degrees and articular gap or step more than 2 millimeters as indications for surgical intervention but the evidence level is moderate [7]. 

One important indication is comminution for plating but it is not exactly determined that comminution results is most helpful in which site (Medial, lateral, anterior, posterior or a combination of them) in more instability and in the type of fracture plating. The plate can be applied anteriorly, posteriorly and or on both sides [5].

At first, posterior plates were introduced for dorsally displaced fractures to buttress fracture site. With a mechanical view, dorsally angulated fractures are better controlled with dorsal buttress plates. But there were many soft tissue issues and tendon irritation with posterior ones [6]. These plates are used anteriorly after invention of locking plates to prevent dorsal displacement of distal fragments [8]. Although, biomechanical studies show that the number of distal and proximal screws may not necessarily increase the device stability [9]. 

Prolonged immobilization can cause poor functional outcomes despite better rate of union and less rate of reduction loss [3]. Unexpectedly, radiologic and functional results were equal to other plating techniques [10-13]. Metaphyseal comminution is considering as an indication for plating. It is not cleared that comminution in which part is more important and causes more instability. In different studies, various treatment methods had equal results and distal radial fractures which introduced as the most controversial fractures [7]. In Moss *et al*., [14] study, the effects of using 7 screws to fix distal radial fractures was compared with using 4 screws on stability of fracture sides. The results showed that increasing in the number of screws from 4 to 7 did not influence on initial stiffness and higher failure loads in fracture sides [14]. Sobky *et al*., [15] showed that volar fixation of unstable distal radial fractures with a fixed angle device is a reliable method to stabilize the fracture sides. There is no study which compares anterior and posterior platting approach in distal fractures of radius. 

If plating provides high stability and fixation in comminuted distal radial fractures and in which comminution sites, this biomechanical study aims to determine that the plates are more helpful. The present study will compare biomechanical benefits of anterior and posterior plating. 

## Materials and Methods

This research was an experimental study which was done based on CT scan images of normal subjects. CT scan images of 10 normal subjects were selected in this study. An ethical approval was obtained from the Ethical Committee of Shiraz University of Medical Sciences.

In this research, the stress were developed in fractured parts and the relative movement of fractured bones were analyzed. Moreover, the stress were developed in various parts of the implant include the implant body and screws were analyzed.

**Fig. 1:**
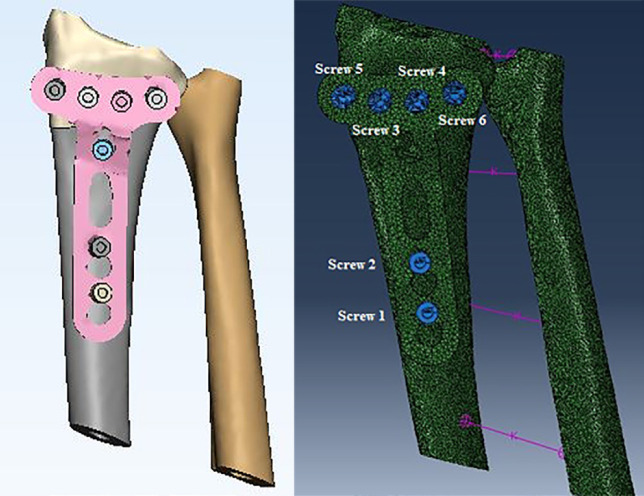
The model developed of writ structures in ABAQUS and 3-Matic

In this study, the distal part of radius was fractured and two configurations of the implants were selected. Actually, in the present study, 2 different models were developed based on CT scan images which includes wrist joint model with a fracture at the distal part and with an implant inserted on the volar surface and wrist joint model with a fracture at the distal part with an implant (with tie screws) on the dorsal surface (Figure 1).

**Fig. 2:**
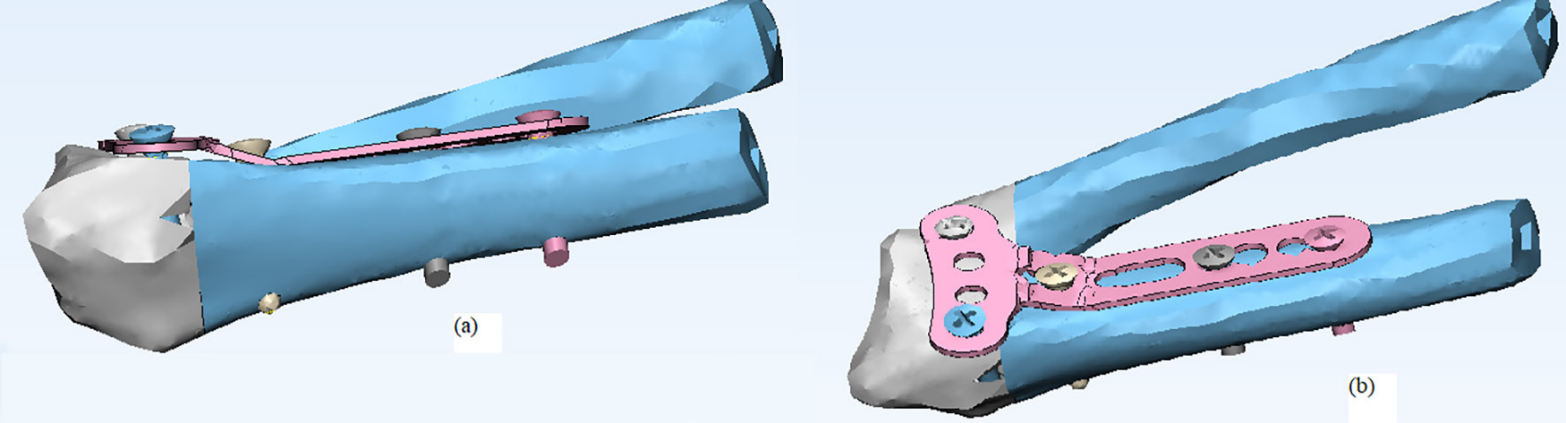
The model of fracture of distal part of radius in 3 Mat software, a=lateral view, b= superior view

It should be emphasized that the analysis was done for flexion and extension forces separately. Figure 2 shows the models of Mimics and Abaqus used in this study.

As it was mentioned before, CT scan images of wrist, radius and ulna were used to create the 3D model of forearm. The developed model consists of radius, ulna and interosseous membrane and ligaments which connected both radius and ulna together. CT scan images had a slice thickness of 1.5 mm and a resolution of 0.098 mm. The CT scan images were from hand (up to MCP joints), wrist, ulna and radius (up to one third of proximal parts). The images were from frontal, sagittal and transverse planes. 

3d modeling was done by using of Mimics software (Materialize interactive medical image control system) version 19 for research produced by Materialized Company, Belgium.

It was done based on the following steps:

1. Thresholding based on the Hounsfield limit.

2. Splinting the segments into separate parts (radius and ulna).

3. Creating 3D models based on the generated region mask.

4. Use of 3-Matic software to mesh the parts and to change the format of the parts from surface to volume.

Mesh Preparation

**Fig. 3:**
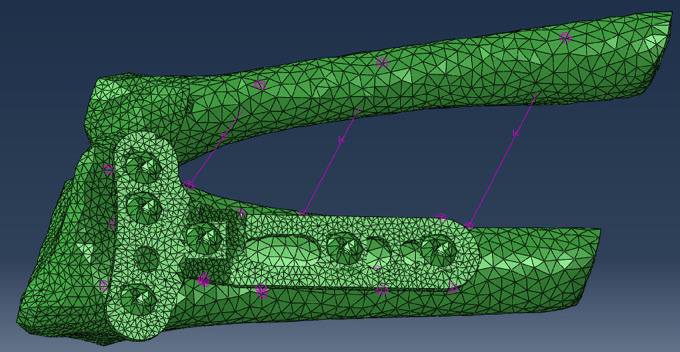
The model of wrist joint in Abaqus with supportive ligaments

The 3D model of the wrist joint (ulna and radius) were exported from Mimics to 3-Matic software (version 19 for research Materialized company Belgium) (Figure 3). The format of the mesh was also changed from three to the teeth and from surface mesh to volume mesh. In the next stage, the mesh was optimized based on the ratio of the length side to minimum length side of triangle element (it should be no longer than 10, its minimum interior angle should be more than 20 degrees and maximum interior angle should be less than 120 degrees).

Re-meshing of the model was done based on the following steps:

1. Remove of the sharp triangle

2. Reduce the total number of triangle

3. Optimize triangular shapes and create the uniform mesh

4. Reduce small triangle

5. Volumetric mesh 

Material Assignment and Modeling of Ligaments Structure

The bone mineral density of radius and ulna was obtained from literature and was applied to the model [6, 16-18]. The supportive ligament consisted of interosseous ligaments with the stiffness of 60 N/mm [19-22].

Boundary Condition and Force Assignments

The proximal ends of radius and ulna were selected as a boundary condition in this research.

The flexion and extension forces (40 N flexion and extension force) were applied on the upper and lower parts of the distal articular surface of radius. It should be emphasized that the force of flexion and extension applied separately on the model. The distal part of radius was fractured in 3-Matic software. The implant was inserted on the volar and dorsal surfaces of radius and was attached to bone by using of screws. 

Some parameters were selected in this study such as the stress applied on proximal and distal parts of fractured radius, stress applied on the implant and screws, the relative displacement of implant and fracture segments, and displacement of screws. The difference between the mean values of these parameters was evaluated in different models by two-sample t-test. Figures 1 and 2 show some procedure of this study.

## Results

The Von Mises stress (MPascal) mean values of different bones, implant and attachment screws follow the extension are shown in Table 1. The maximum stress values developed in the implant (inserted on the volar surface) was 7.77×10^-15^±1.24×10^-11^ compared to 4.46×10^-9±^6.36×10^-10^ M-pa for the dorsal implant (*p*≤0.0010). The implant location did not influence on the stress developed in ulna. The stress developed in proximal and distal parts of the radius differs significantly between the implants inserted on volar and dorsal surfaces. The stress developed in screws was also evaluated in this study. The stress of the screw varied between 0.126 and 19×10^-2 ^M-pa for the implant inserted on the volar surface, compared to 0.16-0.29 M-pa for the dorsal implant (*p*≤0.0010).

**Table 1:** The mean values of the stress developed in various parts of bones and implant when extension force applied

	**Volar**	**Dorsal**	*P* **-value**
Implant	7.77×10^-15^±1.24×10^-11^	4.46×10^-9^±6.36×10^-10^	<0.001
Ulna	49.52±2.35	49.73±2.48	0.43
Radius Distal	107.16±3.84	25.8±2.5	<0.001
Radius Proximal	50.87±1.98	57.14±165	<0.001
Screw 1	0.291±2.66×10^-2^	0.46±0.02	<0.001
Screw 2	0.290±4.77×10^-3^	0.17±0.013	<0.001
Screw 3	0.161±2.61×10^-3^	0.16±0.019	<0.001
Screw 4	0.186±6.09×10^-3^	0.3±0.02	<0.001
Screw 5	0.126±9.51×10^-3^	0.3±0.02	<0.001
Screw 6	0.128±2.83×10^-3^	0.27±0.023	<0.001

The displacements of the implant structure (implant and attachment screws) are shown in Table 2. The total implant displacement was 3.97×10^-2^±3.07×10^-3^ mm for volar plate and 6.79×10^-3^±<0.001058 mm for the dorsal plate, respectively (*p*≤0.0010). There was a significant difference between the displacement of radius parts (proximal and distal parts), screw 1-6 between the implants inserted on volar and dorsal surfaces (follow application of extension force).

**Table 2:** The mean value of the displacement of various parts of bones and implant when extension force applied

	**Volar**	**Dorsal**	***P*** **-value**
Implant	3.97×10^-2^±3.07×10^-3^	6.79×10^-3^±<0.001058	<0.001
Radius Distal	2.69×10^-2^±5.17×10^-3^	1.97×10^-2^±<0.00185	<0.001
Radius Proximal	2.05×10^-2^±3.15×10^-3^	2.84×10^-3^±<0.001066	0.02
Screw 1	3.05×10^-5^±1.39×10^-5^	1.66×10^-2^±<0.00117	<0.001
Screw 2	2.81×10^-5^±8.06×10^-6^	3.42×10^-5^±5.1×10^-6^	0.07
Screw 3	4.30×10^-3^±5.32×10^-4^	6.89×10^-3^±55×10^-5^	<0.001
Screw 4	4.34×10^-3^±8.44×10^-4^	9.53×10^-3^±1×10^-3^	<0.001
Screw 5	5.16×10^-3^±1.04×10^-3^	6.13×10^-3^±37×10^-5^	0.07
Screw 6	4.27×10^-3^±9.56×10^-4^	8.96×10^-3^±4×10^-3^	<0.001

The stress developed in the various parts and their displacement when flexion force applied to the system are shown in Tables 3 and 4.

**Table 3:** The mean value of the stress developed in various parts of bones and implant when flexion force applied

	**Volar**	**Dorsal**	***P*** **-value**
Implant	2.53×10^-9^±3.56×10^-10^	7.24×10^-10^±9.5×10^-11^	<0.001
Ulna	49.52±2.35	49.73±2.2	0.43
Radius Distal	23.73±1.55	106.914±6.15	<0.001
Radius Proximal	50.47±2.16	53.27±2.51	0.046
Screw 1	0.39±1.46×10^-2^	0.32±0.026	<0.0011
Screw 2	0.171±1.28×10^-2^	3×10^-1^±<0.00192	<0.001
Screw 3	0.144±7.34×10^-3^	1.82×10^-1^±0.011	<0.001
Screw 4	0.190±1.50×10^-2^	2×10^-1^±<0.00176	0.054
Screw 5	0.199±1.91×10^-2^	1.78×10^-1^±0.025	0.09
Screw 6	0.177±8.10×10^-2^	2.24×10^-1^±0.016	0.057

**Table 4:** The mean value of the displacement of various parts of bones and implant when flexion force applied

	**Volar**	**Dorsal**	***P*** **-value**
**Implant**	5.56×10^-3^±6.03×10^-4^	4.97×10^-2^±<0.00178	<0.001
Radius Distal	1.59×10^-2^±3.78×10^-3^	3.35×10^-2^±<0.00155	<0.001
Radius Proximal	2.67×10^-3^±5.87×10^-4^	2.78×10^-2^±<0.00145	<0.001
Screw 1	1.82×10^-2^±5.13×10^-3^	2.57×10^-5^±6.95×10^-6^	<0.001
Screw 2	3.14×10^-5^±2.67×10^-6^	3.68×10^-5^±5.84×10^-6^	0.07
Screw 3	6.43×10^-3^±4.62×10^-4^	4.82×10^-3^±36×10^-5^	<0.001
Screw 4	7.91×10^-3^±1.10×10^-3^	4.44×10^-3^±49×10^-5^	<0.001
Screw 5	6.20×10^-3^±5.40×10^-4^	5.06×10^-3^±12×10^-4^	0.07
Screw 6	8.25×10^-3^±7.40×10^-4^	6.15×10^-3^±52×10^-5^	<0.001

As shown in Table 3, the implant position (whether on volar or dorsal surfaces) did not influence on stress developed in ulna. The stress developed in distal parts of radius were 23.73±1.55 and 106.91±6.15 M-pa for volar and dorsal implants, respectively (*p*≤0.0010). However, the stress developed in the proximal part of the radius differed significantly if the implant attached on the volar or dorsal surface (*p*≤0.0010). The stress of screws 1-3 were increased significantly in implant inserted on the dorsal surface compared to the volar surface. However, it did not differ significantly for screw 4-6.

As shown in Table 4, the displacement mean value of the implant attached on the volar surface was significantly less than the implant attached on the dorsal surface (*p*≤0.0010).

In contrast, the distal part of the radius were moved nearly less than half in the implant attached to the dorsal surface compared to the value surface. The proximal part of the radius moved by 2.67×10^-3^±5.87×10^-4 ^ mm in the volar plate condition compared to 2.78×10^-2^±<0.00145 mm in dorsal plate. The first screw displacement was significantly more in volar plate inserted than the dorsal plate (1.82×10^-2^±5.13×10^-3^ and 2.57×10^-5^±6.95×10^-6 ^mm, respectively).

## Discussion

The distal radius fractures incidence are high due to road traffic accidents (RTA) in younger patients and due to low energy traumas in the elderly subjects. The purpose of treatment in this kind of fractures are stability in fracture parts, early rehabilitation, and mobilization. Surgical approach were used for this group of the subjects and using of plate is one of the recommended treatment. The aim of this study was to evaluate the stress developed in various parts of fractured and implant structures inserted on volar and dorsal surfaces. Moreover, it was aimed to determine the displacement of various parts of bones and implant in two different conditions of the implant insertion.

As it was mentioned, the implant was evaluated while flexion and extension forces are applying to the forearm model. As shown before, the stress applied to both bony and implant structures is significantly more for the dorsal inserted implant following by extension force applied. However, it is not too high to deform the structures especially the stress of implant and screws is too low compared to the yield stress of the structure [23]. Bony displacement structure and implant showed that the displacement is too small for extension force and it is significantly different between conditions. The screws displacement is higher in dorsal implant than volar implant. However, the implant stress of bone (proximal and distal parts) and implant significantly were increased in volar inserted implant. Although these movements are too small, they may increase the incidence of tendon irritation.

The stress and displacement of body parts fractures and implants show that the stress of both bony structure and implant parts were increased significantly for the implant inserted on the dorsal surface. However, the stress is low compared to maximum stress which can be tolerated by both bony and implant structures.

The displacement of implant, radius parts and screw1 was more in the dorsal surface than implant inserted on the volar surface. However, the displacement were increased in the volar inserted implant for the other screws. As it was mentioned before, although the displacement of body structure and implants are too small; however, they may increase the chance of tendon irritation. Based on the results of this study, although the location of the implant influence the stress developed in the structure (it was mostly high in dorsal implant compared to the volar implant), the stress is not too high to fail the structure [23].Therefore, it can be concluded that both types of approaches provide enough stability in fracture site based on the displacement of bony structure and implant and stress developed and their stability is not influenced by flexion or extension forces.

The important point which should be considered is the displacement of bony and implant structures. As it was mentioned, the displacement of the implant and bony parts is more in volar inserted than in the main implant body; however, it was increased mostly in the screws for the dorsal inserted implant. It seems that the irritation of soft tissue, tendon and muscles mostly occur due to movement of screws. If it is the irritation cause, the incidence of tendon tear and irritation with volar inserted plate is less than dorsal inserted plate. Therefore, it can be concluded that the side effects associated with volar plate is less than with dorsal plate.

One important clue could be using of screws lock which move less than ordinary cortical non-locking screws in case of dorsal plate application to reduce soft tissue irritation. It recommends to use locking plates which could be more necessary in dorsal plates. Typical compression plates may be enough in volar approach as they are significantly less expensive and more accessible**.**

The results of this study confirmed that although the stress applied on bone and implant structures differed between dorsal and volar inserted conditions, the stress is not too high to fail the structure. The displacement of fractured parts and implant is more in volar plate than dorsal plate. However, screws move in dorsal plate more than volar plate. As far as the irritation of soft tissue be considered, the volar plate feasibility seems to be more than dorsal plate.

## Ethics Approval:

An ethical approval was obtained from the Ethical Committee of Shiraz University of Medical Sciences. 

## Funding Support:

Shiraz University of Medical Sciences.

## Conflict of Interest:

None declared.
